# Preservation of microbial communities enriched on lignocellulose under thermophilic and high-solid conditions

**DOI:** 10.1186/s13068-015-0392-y

**Published:** 2015-12-02

**Authors:** Chaowei Yu, Amitha P. Reddy, Christopher W. Simmons, Blake A. Simmons, Steven W. Singer, Jean S. VanderGheynst

**Affiliations:** Department of Biological and Agricultural Engineering, University of California, One Shields Ave, Davis, CA 95616 USA; Joint BioEnergy Institute, Emeryville, CA 94608 USA; Department of Food Science and Technology, University of California, Davis, CA 95616 USA; Biological and Materials Science Center, Sandia National Laboratories, Livermore, CA 94551 USA; Earth Sciences Division, Lawrence Berkeley National Laboratory, Berkeley, CA 94720 USA

**Keywords:** Biological lignocellulose deconstruction, Cryopreservation, Microbial community enrichment

## Abstract

**Background:**

Microbial communities enriched from diverse environments have shown considerable promise for the targeted discovery of microorganisms and enzymes for bioconversion of lignocellulose to liquid fuels. While preservation of microbial communities is important for commercialization and research, few studies have examined storage conditions ideal for preservation. The goal of this study was to evaluate the impact of preservation method on composition of microbial communities enriched on switchgrass before and after storage. The enrichments were completed in a high-solid and aerobic environment at 55 °C. Community composition was examined for each enrichment to determine when a stable community was achieved. Preservation methods included cryopreservation with the cryoprotective agents DMSO and glycerol, and cryopreservation without cryoprotective agents. Revived communities were examined for their ability to decompose switchgrass under high-solid and thermophilic conditions.

**Results:**

High-throughput 16S rRNA gene sequencing of DNA extracted from enrichment samples showed that the majority of the shift in composition of the switchgrass-degrading community occurred during the initial three 2-week enrichments. Shifts in community structure upon storage occurred in all cryopreserved samples. Storage in liquid nitrogen in the absence of cryoprotectant resulted in variable preservation of dominant microorganisms in enriched samples. Cryopreservation with either DMSO or glycerol provided consistent and equivalent preservation of dominant organisms.

**Conclusions:**

A stable switchgrass-degrading microbial community was achieved after three 2-week enrichments. Dominant microorganisms were preserved equally well with DMSO and glycerol. DMSO-preserved communities required more incubation time upon revival to achieve pre-storage activity levels during high-solid thermophilic cultivation on switchgrass. Despite shifts in the community with storage, the samples were active upon revival under thermophilic and high-solid conditions. The results suggest that the presence of microorganisms may be more important than their relative abundance in retaining an active microbial community.

**Electronic supplementary material:**

The online version of this article (doi:10.1186/s13068-015-0392-y) contains supplementary material, which is available to authorized users.

## Background

Development of economical and sustainable conversion technologies for production of lignocellulosic biofuels is needed to meet the one billion gallon renewable fuel requirement set in the 2013 Clean Air Act [1–5]. Lignocellulosic biomass feedstocks, such as agricultural residues and dedicated energy crops, are primarily composed of complex matrices of polysaccharides (cellulose and hemicellulose) and lignin which form plant cell walls [3]. These complex plant cell walls are recalcitrant to biological conversion, resulting in a high cost for pretreatment and enzymatic hydrolysis [3, 6–8]. The discovery of efficient and economically viable enzymes and microorganisms for use at the industrial scale would significantly lower the production cost of ethanol from lignocellulosic biomass [8–10].

The industrial processes for converting lignocellulosic biomass to liquid fuel typically require enzymes that function at extreme conditions, such as high temperature, high-solid loading, and low moisture [9–11]. Microbial communities found in nature, such as in compost and soil environments, are very efficient at deconstructing lignocellulosic plant biomass and are a potential source of such enzymes. However, compost and soil ecosystems are often too complex for direct identification of deconstructive microorganisms and enzymes. To address this, enrichment culture, where engineered culture conditions are used to promote growth of microorganisms with specific traits, has been applied [11–15]. In enrichment cultures to promote growth of lignocellulolytic microorganisms, biomass feedstocks are used as substrate and complex communities, such as those found in compost or soil are applied as inoculum [11, 16]. Cultures are subjected to thermophilic and high-solid loading conditions to simulate environments relevant to industrial biofuel production. The enrichment process generates less complex lignocellulolytic microbial communities that can facilitate targeted discovery of potential enzymes and microorganisms for biomass deconstruction [11].

Preservation of enriched microbial communities is important for industrial applications and research [17, 18]. For long-term storage of individual microorganisms, cryopreservation and lyophilization are two major methods used; however, cryopreservation is generally preferred over lyophilization due to potential cell damage during the drying process [19]. Proper preservation methods should not change the morphology, physiology and genetics of the organism [18]. To minimize cellular damage during cryopreservation and thawing processes, microorganisms are typically preserved in the presence of cryoprotective agents, such as dimethyl sulfoxide (DMSO) and glycerol [20]. While there have been many studies examining storage of individual microorganisms, few studies have focused on preservation of microbial communities and evaluation of preservation methods using high-throughput DNA sequencing [17, 18, 21].

The goal of this study was to evaluate the composition of microbial communities enriched on switchgrass before and after application of different preservation methods. The enrichments were completed in a high-solid and aerobic environment at 55 °C. Community composition was examined for each enrichment to determine when a stable community was achieved. Preservation methods included cryopreservation with the cryoprotective agents DMSO and glycerol, and preservation without cryoprotective agents. Revived communities were examined for their ability to decompose switchgrass under high-solid and thermophilic conditions.

## Results

### Enrichment of the switchgrass-degrading community

**Fig. 1:**
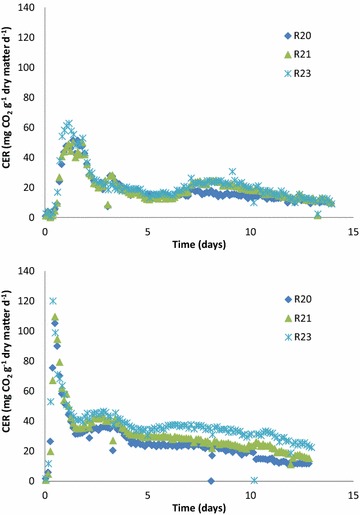
Carbon dioxide evolution rate (CER) profiles for **a** the first (T1) 2 weeks and **b** sixth (T6) 2 weeks of the enrichment study for individual reactors (R20, R21 and R23)

Carbon dioxide evolution rate (CER) profiles for incubations T1 and T6 are shown in Fig. 1. In the initial enrichment period, respiration rate increased rapidly at the beginning of incubation, reached a peak activity at approximately 1.3 days with an average rate of 55 mg CO_2_ g^−1^ dry matter day^−1^, and then dropped rapidly (Fig. 1a). A second peak in respiration occurred on day 3 and a third peak was observed on day 8; these two peaks were consistent with the watering and mixing schedule (every 3–4 days).

In successive enrichments, the first peak in activity tended to occur sooner during incubation. For T6 the first peak occurred at 9.6 h (Fig. 1b). A second peak was also observed for the T6 enrichment at around 3 days. In the respiration profile for T6, the CO_2_ evolution rate at the first peak was around 110 (mg CO_2_ g^−1^ dry matter day^−1^), which was 2 times greater than the initial peak for T1. The higher peak respiration indicates higher metabolic activity, suggesting that the community in T6 was better adapted for switchgrass decomposition than the community in T1.

The improved ability of the microbial community to decompose switchgrass with each enrichment was also assessed by measuring cumulative respiration (cCER). Different time periods were examined based on peaks observed in the respiration profiles (Fig. 1). During the first 3 days of incubation when the rate of microbial activity was the greatest, average cCER increased with each enrichment (Table 1); the 3-day cCER for T6 was significantly higher than for T1. The cumulative CO_2_ evolution rates (cCER) for each reactor at the end of each 5- and 12.7-day incubation period are also provided in Table 1. The 12.7-day cCER for T6 was significantly higher than T1 (*p* < 0.05), but the 12.7-day cCER values for T3, T4, T5, and T6 were not significantly different, suggesting that the community was likely stabilized between T3 and T6 enrichments.

**Table 1:** Cumulative carbon dioxide evolution rate (cCER) for each enrichment

	Mean cCER (mg CO_2_ g^−1^ dry matter)^a^		
	3-day	5-day	12.7-day
T1	90 (7) A	128 (8) A	258 (24) A
T2	98 (9) A,B	147 (11) A,B	280 (16) A
T3	119 (7) C,D	176 (10) C	312 (25) A,B
T4	113 (14) B,C	160 (26) C	293 (67) A,B
T5	119 (7) C,D	168 (11) C,D	294 (29) A,B
T6	136 (10) D	204 (21) D	400 (71) B

^a^Standard deviation in parenthesis (*n* = 3). Means followed by the same letter within incubation periods are not statistically different at *α* = 0.05 based on Tukey–Kramer HSD test, blocked by reactor

**Fig. 2:**
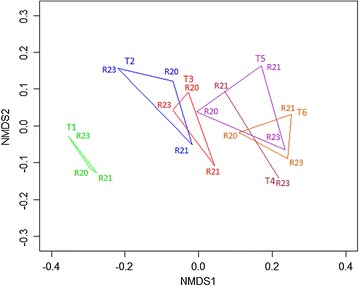
Non-metric multidimensional scaling plot of microbial communities grouped by enrichment time points. Three replicates are shown for all time periods except T4 samples which had 2 replicates

**Fig. 3:**
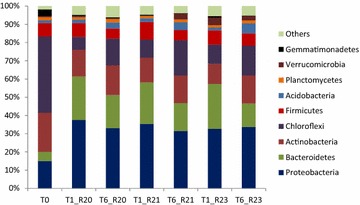
Relative abundance of phyla in communities at enrichment time T0, T1, and T6

The microbial community shifted in structure over the course of enrichment (Fig. 2). The T1 microbial communities for the three replicated reactors were similar; however, as enrichment progressed, each replicate tended to shift to a unique microbial community structure. Shifts in phylum abundance were also observed (Fig. 3; Table 2). The dominant phyla included *Chloroflexi, Proteobacteria, Bacteroidetes* and *Actinobacteria. Chloroflexi* was the most abundant (42 %) phylum in the compost inoculum (T0), dropped to 7–10.5 % in the initial enrichment T1, and significantly increased to 15–19 % in T6. Between T0 and T1, the relative abundance of *Bacteroidetes* increased from 5 % up to 24 %, and significantly decreased to 13–18 % by T6. Abundance levels of *Proteobacteria* increased from an initial 15 up to 35 % after the first 2 weeks of enrichment and remained relatively constant. The relative abundance of *Actinobacteria* decreased between T0 and T1 and remained relatively constant during the remaining enrichments. Similar trends were observed in all three reactors.

**Table 2:** Mean relative abundance (%) of dominant phyla in communities T1 and T6

	Acidobacteria	Actinobacteria	Bacteroidetes	Chloroflexi	Firmicutes	Planctomycetes	Proteobacteria	Verrucomicrobia	Gemmatimonadetes	Other phyla
T1	1.84 A	13.11 A	23.75 A	9.18 A	8.34 A	1.27 A	35.22 A	1.73 A	0.61 A	4.96 A
T6	4.39 B	15.66 A	15.41 B	16.78 B	5.94 B	1.73 B	32.76 A	2.03 A	0.29 A	5.03 A

Means followed by the same letter within columns are not statistically different at *α* = 0.05 based on Tukey–Kramer HSD test

**Fig. 4:**
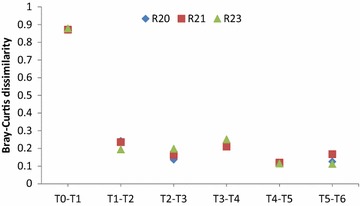
Bray–Curtis dissimilarity values for communities compared at different enrichment times

The microbial community diversity decreased between T0 and T1 and stayed relatively constant between T1 and T6 (Fig. 4). The richness of the communities decreased after 2 weeks of enrichment (T0 to T1), continued to decrease slowly between T1 and T3, and became stable after T3 (Table 3). The evenness of the communities in all reactors dropped after the first 2-week enrichment but gradually increased with later enrichments. The evenness in R20 became steady after T3 while both R21 and R23 continued to increase after T3. Bray–Curtis dissimilarity values for all three reactors were close to 1 when comparing the communities at time T0 and T1, indicating that the initial community structure (T0) and the community structures after the first 2 weeks of enrichment (T1) were very different (Fig. 4). However, as enrichment progressed from T1 to T6, Bray–Curtis dissimilarity values for all three reactors decreased and stayed relatively stable with the lowest values observed between T4 and T5. The analyses of richness, evenness and dissimilarity suggest that a relatively stable switchgrass-degrading community was achieved by enrichment T3 (Table 3).

**Table 3:** Mean Shannon diversity, richness, Pielou’s evenness and Bray–Curtis dissimilarity values for microbial communities by enrichment times

	Shannon diversity	Richness	Evenness	Bray–Curtis dissimilarity comparisons to T1
T0	4.20	790	0.63	0.87
T1	3.18 A	268 A	0.57 A	0
T2	3.06 A	192 B	0.58 A,B	0.22 A
T3	3.02 A	160 C	0.59 A,B	0.26 A,B
T4	3.07	154	0.61	0.33
T5	3.07 A	156 C	0.61 B	0.32 B
T6	3.11 A	155 C	0.62 B	0.33 B

Means followed by the same letter within columns are not statistically different at *α* = 0.05 based on Tukey–Kramer HSD test, blocked by reactor. Time point 4 was excluded from the statistical analysis due to insufficient DNA in one sample. T0 included only one sample

SIMPER analysis revealed that *Sphingobacteriales* was the largest contributor to dissimilarity between T1 and T6 enrichment time points for all three reactors (Table 4); its relative abundance decreased with enrichment. Other organisms that contributed to dissimilarity in all reactors included *Anaerolineae* and *Micromonosporaceae*, which both increased with enrichment.

**Table 4:** OTUs that contribute >4 % to Bray–Curtis dissimilarity between T1 and T6 communities

Reactor	Abundance in T1 sample (%)	Abundance in T6 sample (%)	% Contribution to dissimilarity	OTU classification^a^
R20	23.59	17.96	9.91	*g__Sphingobacteriales*
R20	2.62	7.80	9.11	*c__Anaerolineae*
R20	2.92	7.69	8.40	*g__Micromonosporaceae*
R20	6.39	2.88	6.17	*g__Streptosporangiaceae*
R20	2.62	5.55	5.16	*g__Roseiflexales* (OTU 10)
R20	2.39	0	4.21	*g__Balneimonas*
R21	22.49	15.08	11.54	*g__Sphingobacteriales*
R21	0.01	5.94	9.23	*g__Roseiflexales* (OTU 18)
R21	4.23	9.06	7.52	*c__Anaerolineae*
R21	2.60	6.93	6.74	*g__Micromonosporaceae*
R21	6.29	3.27	4.70	*g__Sinobacteraceae*
R23	24.58	12.68	15.88	*g__Sphingobacteriales*
R23	5.17	11.02	7.80	*c__Anaerolineae*
R23	0.67	6.27	7.47	*g__Micromonosporaceae*
R23	4.17	0.40	5.02	*g__Chthoniobacter*
R23	6.31	2.64	4.89	*g__Streptosporangiaceae*
R23	1.21	4.41	4.27	*g__Candidatus Solibacter*

^a^Letter codes indicate the highest resolved taxonomy from phylogenetic binning: *k* kingdom, *p* phylum, *c* class, *o* order, *f* family, *g* genus

Despite all communities originating from the same inoculum source and identical culture conditions, there were differences in the evolution of the enriched communities (Additional file 1: Figure S1). Comparisons of dissimilarity between enriched microbial communities for each reactor at the final enrichment (T6) showed *Roseiflexales* to be the largest contributor to dissimilarity. Two different species were detected and levels varied between enrichment times and reactors. Very low levels were detected in R23. For R20, relative abundance averaged 6 % for T2–T6, while for R21 relative abundance was low until enrichment T4 at which time levels increased to 7–8 %.

### Influence of storage methods on culture preservation

The cumulative carbon dioxide evolution rates (cCER) for all treatments are shown in Table 5. The cCER levels of reactors measured on day 3 and day 6 show that both −80 °C and DMSO-treated samples had significantly lower activities than control and glycerol-treated samples. The higher activities in glycerol-treated samples were likely due to the presence of glycerol in the re-inoculated feedstock and its potential utilization as a carbon source. The cCER levels at 12.7 days were between 311 and 388 (mg CO_2_ g^−1^ dry matter). At 12.7 days there were no significant differences between treatments.

**Table 5:** Cumulative carbon dioxide evolution rate (cCER) of control (T7) and stored treatments measured after 3, 6 and 12.7 days of incubation

	Mean cCER (mg CO_2_ g^−1^ dry matter)^b^			Mean ratio^a^		
	3-day	6-day	12.7-day	3-day	6-day	12.7-day
T7	125 (17) AB	250 (66) A	369 (97) A	0.91 A	1.06 A	0.96 A
−80 °C	97 (31) BC	156 (34) B	311 (36) A	0.70 B	0.66 B	0.83 A
DMSO	91 (15) C	161 (22) B	388 (31) A	0.66 B	0.69 B	1.07 A
Glycerol	130 (34) A	228 (58) A	384 (70) A	0.94 A	0.97 A	1.02 A

^a^Ratio of (cCER post storage)/(cCER of T6)

^b^Standard deviation in parenthesis (*n* = 3). Means followed by the same letter within columns and incubation periods are not statistically different at *α* = 0.05 based on Tukey–Kramer HSD test, blocked by reactor

The cCER of each treatment was also examined relative to the cCER of the inoculum (T6). The comparisons were calculated as ratios (Table 5). In general, the ratios of both control and glycerol-treated samples were greater than −80 °C and DMSO-treated samples, indicating that both −80 °C and DMSO may not have preserved the active microbial community responsible for switchgrass decomposition. However, for DMSO-treated samples, the ratio was relatively low on day 3 and 6 but increased toward the end of the incubation. It is possible that the DMSO-treated community required a longer time to acclimate to the growth environment compared to communities preserved in glycerol.

**Fig. 5:**
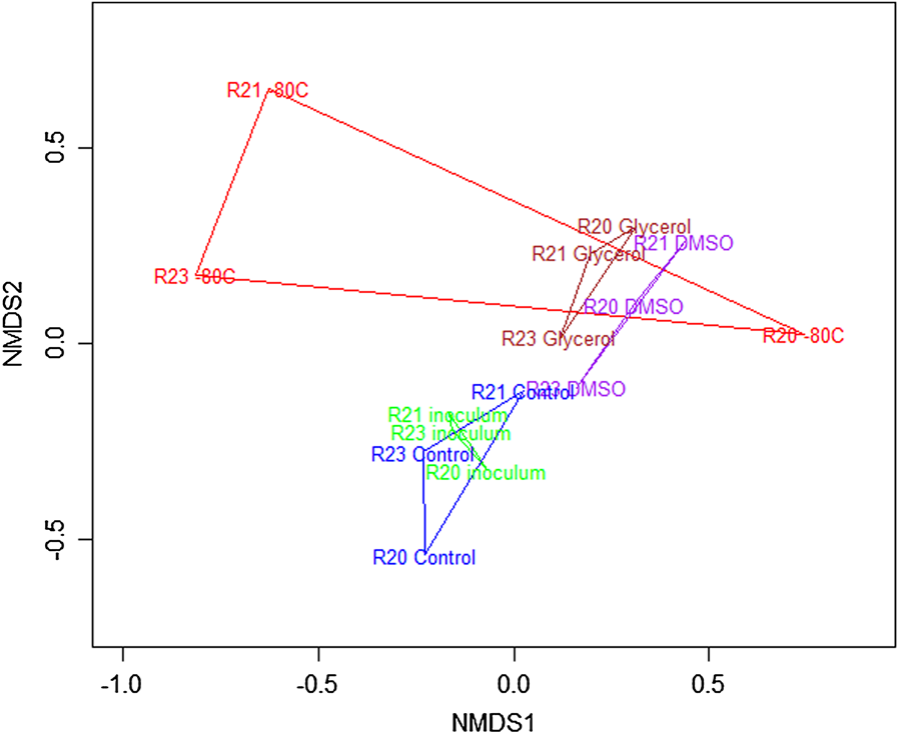
Non-metric multidimensional scaling plot of microbial communities grouped by storage methods

The NDMS plot indicates that there was a shift in the community structure of the stored microbial communities upon inoculation and incubation on switchgrass (Fig. 5). The largest shift occurred with −80 °C storage in the absence of cryoprotectant. Unlike DMSO and glycerol-treated communities, which tended to cluster together on the NDMS plot, each of the reactor communities diverged during storage at −80 °C. The results indicate −80 °C storage in the absence of cryoprotectant would provide inconsistent preservation of thermophilic communities enriched on switchgrass. For this reason, samples from this treatment were not considered for further comparisons.

The microbial community of the inoculum and control samples (T7) had equivalent values for diversity, richness and evenness (Table 6). In general, values for these diversity indices decreased with preservation. The richness of samples preserved with DMSO and glycerol was significantly smaller than inoculum (T6) and T7 samples. Bray–Curtis dissimilarity values for inoculum communities compared to control communities were small (<0.34), indicating that the inoculum community structures and control community structures were similar (Table 6). Bray–Curtis dissimilarity values were greater for DMSO- and glycerol-treated samples indicating both preservation methods altered the community structure present in the inoculum. The greatest differences were observed for the glycerol-treated community.

**Table 6:** Mean Shannon diversity, richness, Pielou’s evenness and Bray–Curtis dissimilarity values for microbial communities from different treatments

	Shannon diversity^a^	Richness^a^	Evenness^a^	Bray–Curtis dissimilarity comparison to inoculum^a^
Inoculum	3.11 A,B	155 A	0.62 A,B	0
Control	3.20 A	160 A	0.63 A	0.21 A
DMSO	2.71 B	131 B	0.56 B	0.39 A,B
Glycerol	2.83 A,B	132 B	0.58 A,B	0.41 B

^a^Means followed by the same letter within columns are not statistically different at *α* = 0.05 based on Tukey–Kramer HSD test, blocked by reactor

The relative abundances of several phyla were affected by storage treatments (Table 7). *Actinobacteria* and *Firmicutes* were both resilient to cryopreservation; the relative abundance of these phyla significantly increased with treatment. The relative abundances of *Chloroflexi* and *Planctomycetes* in DMSO and glycerol-treated samples were significantly lower than the control and inoculum samples. *Acidobacteria*, *Bacteroidetes*, *Proteobacteria* and *Verrucomicrobia* did not change with preservation.

**Table 7:** Mean relative abundance (%) of phyla in communities

	Acidobacteria	Actinobacteria	Bacteroidetes	Chloroflexi	Firmicutes	Planctomycetes	Proteobacteria	Verrucomicrobia	Other phyla
Inoculum	4.39 A	15.66 A	15.41 A	16.78 A	5.94 A	1.73 A	32.76 A	2.03 A	5.31 A
Control	4.81 A	17.89 A	9.27 A	15.3 A	6.60 A,B	1.60 A,B	38.82 A	1.48 A	4.20 A,B
DMSO	6.11 A	30.23 B	17.20 A	4.75 B	11.35 B,C	0.24 C	27.02 A	0.99 A	2.11 A,B
Glycerol	4.56 A	24.92 B	17.55 A	3.46 B	11.75 C	0.63 B,C	34.69 A	1.13 A	1.30 B

Means followed by the same letter within columns are not statistically different at *α* = 0.05 based on Tukey–Kramer HSD test, blocked by reactor (*n* = 3)

Very small changes in relative abundance were observed between the inoculum and control communities. SIMPER analysis indicated that *Sphingobacteriales* was a large contributor to dissimilarity between T6 and T7 enrichment time points (Table 8). The abundance of *Sphingobacteriales* decreased slightly with additional enrichment between the inoculum (T6) and control (T7).

**Table 8:** OTUs that contribute >4 % to Bray–Curtis dissimilarity between inoculum communities (T6) and control (T7) communities

Reactor	Abundance in T6 sample (%)	Abundance in T7 sample (%)	% Contribution to dissimilarity	OTU classification^a^
R20	17.96	5.45	18.19	*g__Sphingobacteriales*
R20	5.55	0.84	6.85	*g__Roseiflexales* (OTU 10)
R20	0.06	4.76	6.83	*g__Alkalilimnicola*
R20	5.57	1.08	6.54	*k__Bacteria*
R20	0.21	4.11	5.66	*g__Rhizobiales*
R20	1.52	5.13	5.25	*g__Pseudoxanthomonas*
R21	6.93	15.39	22.55	*g__Micromonosporaceae*
R21	15.08	9.41	15.13	*g__Sphingobacteriales*
R21	3.93	7.51	9.55	*g__Chelatococcus*
R21	5.94	3.32	6.97	*g__Roseiflexales* (OTU 18)
R21	3.21	1.00	5.89	*g__Streptosporangiaceae*
R23	11.02	13.05	10.98	*c__Anaerolineae*
R23	6.27	4.49	9.64	*g__Micromonosporaceae*
R23	4.83	3.21	8.75	*g__Chelatococcus*
R23	0.42	1.53	5.99	*g__Roseiflexales* (OTU 18)
R23	1.44	0.38	5.71	*g__Rhizobiales*
R23	4.72	5.54	4.46	*k__Bacteria*
R23	0.77	1.59	4.46	*g__Schlegelella*

^a^Letter codes indicate the highest resolved taxonomy from phylogenetic binning: *k* kingdom, *p* phylum, *c* class, *o* order, *f* family, *g* genus

*Micromonosporaceae*, *Anaerolineae* and *Roseiflexales* contributed >4 % to dissimilarity between the inoculum and DMSO and glycerol treatments for all reactors. *Micromonosporaceae* was the top contributor to dissimilarity between T6 and DMSO and glycerol samples (Tables 9, 10); its abundance increased with storage. *Anaerolineae* and *Roseiflexales* decreased in all treatments.

**Table 9:** OTUs that contribute >4 % to Bray–Curtis dissimilarity between inoculum communities (T6) and DMSO communities

Reactor	Abundance in T6 sample (%)	Abundance in DMSO sample (%)	% Contribution to dissimilarity	OTU classification^a^
R20	7.69	27.96	25.35	*g__Micromonosporaceae*
R20	18.47	7.04	14.29	*g__Steroidobacter*
R20	2.69	8.62	7.41	*g__Chelatococcus*
R20	5.55	0.77	5.98	*g__Roseiflexales* (OTU 10)
R20	5.57	0.80	5.96	*k__Bacteria*
R20	0.38	5.01	5.79	*g__Thermobacillus*
R20	7.80	4.31	4.36	*c__Anaerolineae*
R21	6.93	27.67	20.96	*g__Micromonosporaceae*
R21	17.14	2.06	15.23	*g__Steroidobacter*
R21	1.02	9.20	8.26	*g__Thermobacillus*
R21	9.06	1.57	7.56	*c__Anaerolineae*
R21	15.08	22.51	7.51	*g__Sphingobacteriales*
R21	5.94	0.04	5.96	*g__Roseiflexales* (OTU 18)
R23	6.27	19.91	23.80	*g__Micromonosporaceae*
R23	11.02	4.48	11.42	*c__Anaerolineae*
R23	0.15	5.50	9.33	*g__Brevibacillus*
R23	4.72	1.72	5.24	*k__Bacteria*
R23	4.17	1.64	4.43	*g__Roseiflexales* (OTU 10)

^a^Letter codes indicate the highest resolved taxonomy from phylogenetic binning: *k* kingdom, *p* phylum, *c* class, *o* order, *f* family, *g* genus

**Table 10:** OTUs that contribute >4 % to Bray–Curtis dissimilarity between inoculum communities (T6) and glycerol communities

Reactor	Abundance in T6 sample (%)	Abundance in glycerol sample (%)	% Contribution to dissimilarity	OTU classification^a^
R20	18.47	2.43	17.06	*g__Steroidobacter*
R20	7.69	23.71	17.03	*g__Micromonosporaceae*
R20	2.69	11.05	8.89	*g__Chelatococcus*
R20	7.80	1.12	7.10	*c__Anaerolineae*
R20	0.38	6.79	6.82	*g__Thermobacillus*
R20	5.57	0.35	5.55	*k__Bacteria*
R20	5.55	0.35	5.53	*g__Roseiflexales* (OTU 10)
R21	6.93	22.47	18.20	*g__Micromonosporaceae*
R21	17.14	4.59	14.69	*g__Steroidobacter*
R21	1.02	9.76	10.23	*g__Thermobacillus*
R21	5.94	0.03	6.92	*g__Roseiflexales* (OTU 18)
R21	3.93	8.88	5.79	*g__Chelatococcus*
R21	9.06	4.45	5.39	*c__Anaerolineae*
R23	6.27	16.21	14.83	*g__Micromonosporaceae*
R23	11.02	1.94	13.54	*c__Anaerolineae*
R23	12.68	18.47	8.64	*g__Sphingobacteriales*
R23	4.72	0.97	5.59	*k__Bacteria*
R23	1.58	5.29	5.52	*g__Phyllobacteriaceae*
R23	4.17	0.79	5.04	*g__Roseiflexales* (OTU 10)
R23	1.44	4.65	4.79	*g__Rhizobiales*

^a^Letter codes indicate the highest resolved taxonomy from phylogenetic binning: *k* kingdom, *p* phylum, *c* class, *o* order, *f* family, *g* genus

The abundance of *Chelatococcus* increased after microbial communities were stored in glycerol (data from R23 not shown) and DMSO (data from R21 and R23 not shown). The abundance of *Thermobacillus* in R20 and R21 also increased after microbial communities were stored in DMSO and glycerol.

*Steroidobacteria* was also a top contributor to dissimilarity between T6 and DMSO samples, and between T6 and glycerol samples in R20 and R21. The abundance of *Steroidobacteria* in R20 and R21 decreased after storage with DMSO and glycerol. These findings suggest that DMSO and glycerol storage conditions were not favorable for *Anaerolineae, Roseiflexales* and *Steroidobacter*, but did favor *Micromonosporaceae*, *Chelatococcus*, and *Thermobacillus*.

## Discussion

Experiments were done to determine the number of enrichment passages necessary to achieve a stable microbial community capable of degrading switchgrass under thermophilic and high-solid conditions. In general, enrichment beyond T3 did not significantly affect microbial activity or microbial community structure.

*Sphingobacteria* was the largest contributor to dissimilarity between T1 and T6 enrichment time points for all three reactors (Table 4); levels were approximately 23 % at T1 and declined to approximately 16 % at T6. Certain species within *Sphingobacteria* have been observed in an aerobic soil containing decomposing rice callus under mesophilic conditions [22, 23]. In these studies, the experiments were conducted for 56 days, and *Sphingobacteria* became more dominant with decomposition. *Sphingobacteria* have also been found to positively interact with algal growth in municipal wastewater at room temperature [24]. A gene (*xynA19*) cloned from *Sphingobacteria* sp. TN19 was found in the gut of *Batocera horsfieldi* larvae, and it has been reported to be a xylanase-encoding gene [25]. An earlier study of wheat straw enriched with chicken manure showed that an isolated *Sphingobacteria* was able to metabolize lignin in a thermophilic environment [26]. The relative abundance of *Sphingobacteria* has been reported to increase after the microbial community from sediments was enriched on wheat straw under alkaline (pH 9), anaerobic, and mesophilic conditions [27]. Furthermore, *Sphingobacteria* have been shown to have the ability to deconstruct hemicellulose in wheat straw enriched with soil at 25 °C in aerobic conditions [28, 29]. Clearly, *Sphingobacteria* can survive in both aerobic and anaerobic conditions and in mesophilic and thermophilic environments. In the present study, however, the decrease in relative abundance between T1 and T6 may have been due to adaptation and increase in abundance of other microorganisms in the community.

*Anaerolineae* was the second largest contributor to dissimilarity between T1 and T6 for reactors R20 and R23 and it was the third largest contributor to dissimilarity for reactor R21. *Anaerolineae* have previously been found in anaerobic wastewater treatment processes, rice paddy soil, and a deep terrestrial hot aquifer [30, 31]. *Anaerolinea thermophila* was discovered in a thermophilic (55 °C) anaerobic sludge treating organic wastewater and it was suggested to play a role in degradation of organic compounds such as carbohydrates and amino acids [31–33]. In the present study, the abundance of *Anaerolineae* increased with enrichment time. It is possible that there were anaerobic pockets in the samples and air could not diffuse into the pockets, resulting in the survival of *Anaerolineae*.

*Micromonosporaceae* was the third largest contributor to dissimilarity between T1 and T6 for reactors R20 and R23 (Table 4) and it was the fourth largest contributor to dissimilarity for reactor R21. Two species, *M.aurantiaca* and *M.carbonacea*, have been identified in rice straw compost [34, 35], and *M.carbonacea* was able to break down cellulose, hemicelluloses, and lignin in rice straw composted with chicken manure [34]. Several studies showed that cellulose can be degraded by certain species within *Micromonospora* in thermophilic environments [13, 36, 37]. A prior study also showed that *M. aurantiaca* was stable over a wide temperature range (55–70 °C) [35]. In addition, *Micromonospora spp*. was reported in previous studies to effectively degrade lignocellulosic wastes in aerobic conditions [13, 38, 39]. Similarly, in the present study, the enrichments were carried out in thermophilic and aerobic conditions, and the abundance of *Micromonosporaceae* increased as enrichment time increased.

*Balneimonas* (in the *Bradyrhizobiaceae* family) was one of the notable contributors to dissimilarity (4.21 %) between T1 and T6 in reactor R20 (Table 4). The abundance of *Balneimonas* decreased to 0 % as enrichment time increased from T1 to T6. Similar results were observed in R21 and R23. *Balneimonas* was previously found in the roots of *Pennisetum glaucum* in the Kavango of Namimia, in Arabidopsis soil, and in soil crusts in the Kalahari of southern Africa [40–42]. Prior studies were under very dry conditions, opposite to the humid conditions in the present study suggesting that a dry environment may be more favorable for *Balneimonas*. Also, *Balneimonas* was observed in mesophilic conditions in these prior studies; our findings indicate that a thermophilic environment may not be favorable for the growth of *Balneimonas*.

In the order *Actinomycetales*, the family *Streptosporangiaceae* was a noticeable contributor to dissimilarity between T1 and T6 in all three reactors. Prior studies have shown the growth of *Streptosporangiaceae* in soil, rice compost, sugar cane bagasse compost, coffee hulls composts, and swine manure compost [43–45]. In the present study, the abundance of *Streptosporangiaceae* decreased with enrichment. It is possible that other microorganisms became more dominant with enrichment reducing the relative abundance of *Streptosporangiaceae*.

In cryopreservation, the biophysical changes caused by the transition of water to ice during the cooling period are the main causes of cell damage [46]. The rate of cooling controls the concentration of solution surrounding the cell and, therefore, influences the rate of water transport out of cells during cooling [19]. In cryopreservation, cells are introduced to cryogenic temperatures which results in ice crystals in the suspension medium and within cells. Thus, the osmotic shock can induce disruption of organelles and loss of membrane integrity, and cause cell injuries and death [18, 46–49]. In an ideal cryopreservation process, water transports out of cells rapidly to maintain equal salt concentration between extracellular and intracellular media, ice formation occurs externally to the cells, and internal cell damage is prevented [19, 46, 50, 51]. If the cooling rate is too fast, there is not sufficient time for water to transport from the more dilute intracellular solution to the concentrated extracellular medium, resulting in damage to the cell membrane [19, 46].

Cryoprotectants are typically classified into two categories: penetrating and non-penetrating. Penetrating cryoprotectants are more ideal because they protect the cell by lowering the freezing point of water promoting hydrogen bond formation and vitrification of solvents, and preventing ice crystal formation inside the cells [18, 46, 57]. Both glycerol and DMSO have cell-penetrating ability and are commonly used in cryopreservation of microorganisms [18]. DMSO can penetrate both the cell wall and cytoplasmic membrane within 15 and 30 min while it takes glycerol more than 30 min [19, 20]. Glycerol and DMSO can prevent osmotic shock by decreasing the freezing point of water and biological fluid to a minimum of −46 and −73 °C, respectively [20, 51, 58, 59].

While several prior studies have evaluated the effects of storage conditions, such as temperature and storage time, on changes in microbial community structure [52–54], little work has been done to evaluate the effects of cryoprotective agents on community structure [55, 56]. Ideally, the composition of microbial communities after storage should be the same as their initial states.

The relative abundances of *Acidobacteria*, *Bacteroidetes*, *Proteobacteria* and *Verrucomicrobio* were not affected by the storage methods suggesting that they can be preserved in either DMSO or glycerol at −80 °C. The relative abundances of *Chloroflexi* and *Planctomycetes* stored in DMSO and glycerol-treated samples were lower than the control and inoculum samples. So while DMSO and glycerol are typical cryoprotective agents used to reduce ice formation and thus prevent cell death during the freezing process [60], it is possible they were not effective for all organisms in T6 samples resulting in damage to organisms in phyla such as *Chloroflexi* and *Planctomycetes* during freezing. The relative abundance of *Firmicutes* was higher after storage with glycerol. Similar results were observed for samples from a cow rumen stored in glycerol at −80 °C [56]. In the present study, the relative abundance of *Actinobacteria* increased after storage with glycerol at −80 °C. A similar result was observed for *Actinomycete* strains stored in glycerol at −80 °C for 3 months [55]. The relative abundances of *Firmicutes* and *Actinobacteria* were also higher after being stored with DMSO.

Storage conditions were not favorable for *Anaerolineae, Roseiflexales* and *Steroidobacter*, but did favor *Micromonosporaceae*, *Chelatococcus*, and *Thermobacillus*. A prior study observed that the relative abundance of *Anaerolineae* collected from either drained (60 % water holding capacity) or flooded paddy soil increased up to 3 % after storing at either 4 or −20 °C for 30 days [61]. It is unclear whether the decrease in abundance of *Anaerolineae* in our experiments was caused by use of cryoprotectants or by the low-temperature storage condition in our study. Wang and co-workers also observed the abundances of *Micromonosporaceae* were unchanged before and after storage [61] which is in contrast to our findings. In our study, the increase in relative abundance of *Micromonosporaceae* could have been caused by the decrease in abundance of other microorganisms that did not tolerate preservation treatments. It is unclear if the actual abundance of *Micromonosporaceae* stayed the same before and after storage. One early study mixed dried rice straw with chicken, pig, and cattle feces under thermophilic conditions to create a compost community that was then used to inoculate Whatman filter paper [62]. This enrichment yielded a stable microbial community that included *Thermobacillus*, which remained stable for at least 1 year when stored at −80 °C in a medium [0.1 % yeast extract, 0.5 % peptone, 0.5 % CaCO3, 0.5 % NaCl, and H_2_O (pH 8.0)] with 20 % (v/v) glycerol [62, 63]. In our study, the abundance of *Thermobacillus* increased after storage, suggesting that in addition to temperature and cryoprotectants, storing the community on the enrichment feedstock may be an important feature for long-term storage.

For *Roseiflexales*, *Steroidobacter*, and *Chelatococcus*, published storage methods include adding a cryoprotectant before storage. A study stored *Roseiflexus* at −80 °C without any cryoprotectant [64]. Another study stored *Steroidobacter* in glycerol (10 %, v/v) at −80 °C for long-term storage [65]. One study isolated *Chelatococcus* from a sludge sample and stored the microorganism with 15 % (v/v) glycerol at −70 °C [66]. To the best of our knowledge, no study has evaluated the impact of storage conditions on the viability of these three organisms.

## Conclusions

High-throughput 16S rRNA gene sequencing greatly assisted in elucidating the impact of enrichment and preservation methods on switchgrass-degrading microbial communities. The measurements made on enriched samples indicated that little change in microbial activity and microbial community structure occurred beyond three 2-week enrichments. Proper preservation methods should not significantly alter the composition of microbial communities after preservation. Preservation of samples in the absence of cryoprotectant resulted in variable changes in community composition. Samples preserved with DMSO and glycerol did experience a consistent shift in community composition though dominant microorganisms were retained in the active community. Despite shifts in the community with storage, the samples were active upon revival under thermophilic and high-solid conditions. The results suggest that the presence of microorganisms may be more important than their relative abundance in retaining an active microbial community.

## Methods

### High-solid enrichments

Finished green waste compost was obtained from a commercial facility that composts agricultural residues including tree and vine prunings (Northern Recycling, Zamora, CA). Compost was solar dried and stored at 4 °C until applied as inocula. Switchgrass (*Panicum virgatum L.*) was obtained from the Joint BioEnergy Institute (Emeryville, CA) and it was pretreated as described previously [67]. In summary, to remove water-soluble carbohydrates in switchgrass, dried switchgrass was extracted with water for 2 days followed by ethanol for 1.5 days in a soxhlet extractor. Extracted switchgrass was lyophilized at −50 °C for at least 24 h until the residual solvent evaporated. Samples were stored in zipper lock bags at 4 °C until used in experiments. The compositions of the treated switchgrass have been reported previously [67].

High-solid incubations were conducted as described previously [11, 13]. Bioreactors with a 0.2 L working volume were loaded with 5–11 g dry weight of switchgrass and inocula mixture. Prior to incubation, switchgrass was wetted with minimal media [12] to a moisture content of 400 wt % dry basis [g water (g dry solid)^−1^] and equilibrated at 4 °C overnight. For the initial enrichment in each experiment, wetted switchgrass was inoculated with 10 wt % [g dry compost (g dry solid)^−1^] compost. The experiment was conducted with three replicates (R20, R21, and R23). Every 2 weeks, fresh feedstock was inoculated with 10 wt % [g dry enriched sample (g total dry weight)^−1^] of the enriched community and transferred to a new bioreactor. The enrichment experiment ran for 12 weeks, resulting in a total of six sampling points (T1, T2, T3, T4, T5, and T6).

House compressed air was humidified by bubbling it through distilled water and metered to each bioreactor with polycarbonate rotameters (5–50 mL air min^−1^, Dwyer Instruments, Inc., Michigan City, IN). Air was supplied to each bioreactor at 15 mL min^−1^. Incubator temperature was maintained at 35 °C for 1 day, ramped to 55 °C by increasing the temperature by 5 °C every 6 h, and held at 55 °C for the remainder of the enrichments. To maintain the moisture content, water was added to each bioreactor every 3–4 days and the contents were mixed.

Water was removed from the effluent of each reactor by passing through molecular sieves (3A, beads, 8–12 mesh particle size, Sigma–Aldrich, St. Louis, MO) and then passing through a small amount of indicating Drierite (W. A. Hammond Drierite Co., Ltd., Xenia, OH). Dry effluent from reactors was plumbed to a 16-position switching valve (VICI Valco Instruments, Houston, TX.), which switched positions every 20 min as controlled by a personal computer running LabVIEW software (Version 2011 SP1, National Instruments Corp., Austin, TX). The effluent from the valve was sent to an infrared carbon dioxide (CO_2_) sensor (Vaisala, Woburn, MA) and flow was measured with a thermal mass flow meter (Aalborg, Orangeburg, NY). Carbon dioxide and flow data were recorded by LabVIEW.

### Processing and storage of enriched communities

The biomass in each reactor from the final enrichment (T6) was split into 4 subsamples for evaluating the effect of storage methods on the activity of inoculum. One of the subsamples was used immediately following the T6 enrichment to inoculate fresh feedstock at 10 wt % [g dry enriched sample (g total dry weight)^−1^]. Inoculated feedstock was transferred to a new bioreactor (control) for incubation for 2 weeks at 55 °C.

The remaining subsamples were subjected to the three following preservation methods: (1) 4 g wet weight mixed with 6 g of 7 % DMSO by weight [in distilled-deionized water (DDH_2_O)] and stored at −80 °C, (2) 4 g wet weight mixed with 6 g of 15 % glycerol by weight (in DDH_2_O) and stored at −80 °C, and (3) 4 g wet weight and stored at −80 °C without cryoprotectant. The final concentrations of DMSO and glycerol in preserved samples were 5 and 10 %, respectively. After 3 weeks of storage, fresh feedstock was inoculated with 10 wt % [g dry stored sample (g dry solid)^−1^] of the stored community. Inoculated feedstocks were transferred to bioreactors for incubation for 2 weeks at 55 °C.

### DNA extraction and 16S rRNA gene sequencing

At the end of each enrichment, 9 g wet weight-enriched feedstock was collected from each reactor, frozen in liquid nitrogen, homogenized with an oscillating ball mill (MM400, Retsch Inc., Newtown, PA), and extracted using a CTAB protocol [16]. Isolated DNA was purified to remove residual inhibitors using a DNeasy Blood and Tissue Kit (Qiagen, Venlo, the Netherlands). Three replicates were analyzed for all time periods except for T4 which had only 2 replicates, due to insufficient DNA.

Sequencing of a hypervariable region of the broadly conserved 16S rRNA gene was performed on purified DNA by the United States Department of Energy Joint Genome Institute using the Illumina Miseq platform, as previously described [14, 68]. In summary, a fragment of the 16S rRNA gene was amplified from DNA extracts using PCR. The forward primer was the 515f primer with a 5′ Illumina adapter amended via pad and linker sequences. The reverse primer was the 806r primer with Illumina adapter compliment, barcode, pad and linker sequences amended to the 5′ end [68].

### Data analysis

Respiration data from high-solid incubations were used to calculate CO_2_ evolution rates (CER) and cumulative respiration (cCER) from CO_2_ concentration and volumetric flow rate measurements of reactor effluents, as described previously [69]. CER values were normalized by the dry weight of material in the reactor. cCER values were obtained by integrating CER over time.

Sequences obtained through high-throughput sequencing of isolated DNA were quality trimmed, filtered, assembled and assigned to OTUs using methods described previously [68]. 16S rRNA gene read counts were used to conduct ecological and ordination analyses. Singletons were removed to reduce variability. Operational taxonomic unit (OTU) richness, evenness, and Shannon diversity values were computed in R (version 3.0.2, R Foundation for Statistical Computing, Vienna, Austria) using the Vegan package (https://vpn.lib.ucdavis.edu/,DanaInfo=CRAN.R-project.org+package=vegan).

Non-metric multidimensional scaling (NMDS) of Bray–Curtis dissimilarity values between communities was performed with Vegan’s metaMDS function using 1000 random starts. Similarity percentage (SIMPER) analysis was conducted as described previously [70] to determine which OTUs contribute most to Bray–Curtis dissimilarity between certain communities.

Significant differences between treatments were identified using analysis of variance (ANOVA) and least significant difference with a significance level *α* = 0.05. Data were analyzed using SAS software (Version 9.4, SAS Institute Inc., Cary, NC).

## Additional file

10.1186/s13068-015-0392-y Relative abundance of organisms in enriched communities in R20, R21, and R23 by enrichment time.

## Abbreviations

CER: carbon dioxide evolution rate
cCER: carbon dioxide cumulative respiration
SIMPER: similarity percentages
