# Supplementary material for: Preservation of microbial communities enriched on lignocellulose under thermophilic and high-solid conditions
Source: Biotechnol Biofuels. 2015 Dec 2;8:206. doi: 10.1186/s13068-015-0392-y (PMC4667496; doi:10.1186/s13068-015-0392-y)
Supplement: Supplementary file 1 — 10.1186/s13068-015-0392-y Relative abundance of organisms in enriched communities in R20, R21, and R23 by enrichment time. [file 13068_2015_392_MOESM1_ESM.docx]

**Supplemental Material**

Figure S1a. Relative abundance of g_*Sphingobacteriales* in enriched communities in R20, R21, and R23 by enrichment time.

Figure S1b. Relative abundance of c_*Anaerolineae* in enriched communities in R20, R21, and R23 by enrichment time.

Figure S1c. Relative abundance of g_*Micromonosporaceae* in enriched communities in R20, R21, and R23 by enrichment time.

Figure S1d. Relative abundance of g_*Streptosporangiaceae* in enriched communities R20, R21, and R23 by enrichment time.

Figure S1e. Relative abundance of g_*Roseiflexales* (OTU 18) in enriched communities R21 and R23 by enrichment time.

Figure S1f. Relative abundance of g_*Roseiflexales* (OTU 10) in enriched community R20 by enrichment time.
